# Supplementary material for: Modeling glioblastoma heterogeneity as a dynamic network of cell states
Source: Mol Syst Biol. 2021 Sep 16;17(9):e10105. doi: 10.15252/msb.202010105 (PMC8444284; doi:10.15252/msb.202010105)
Supplement: Supplementary file 5 — Source Data for Figure 3 [file MSB-17-e10105-s001.zip › Figure3A_sourcedata/GSEA_3065/nosigdb_state5.GseaPreranked.1622718435914/OLSEN_ADRENERGIC.html]

Details for gene set OLSEN\_ADRENERGIC[GSEA]

|  || Dataset | state5 |
| Phenotype | NoPhenotypeAvailable |
| Upregulated in class | na\_neg |
| GeneSet | OLSEN\_ADRENERGIC |
| Enrichment Score (ES) | -0.7581574 |
| Normalized Enrichment Score (NES) | -2.4385214 |
| Nominal p-value | 0.0 |
| FDR q-value | 0.0 |
| FWER p-Value | 0.0 |
Table: GSEA Results Summary

  

Fig 1: Enrichment plot: OLSEN\_ADRENERGIC      
 Profile of the Running ES Score & Positions of GeneSet Members on the Rank Ordered List

  

| PROBE | GENE SYMBOL | GENE\_TITLE | RANK IN GENE LIST | RANK METRIC SCORE | RUNNING ES | CORE ENRICHMENT || 1 | LUC7L3 |  |  | 65 | 0.589 | 0.0140 | No |
| 2 | NCAM1 |  |  | 70 | 0.581 | 0.0345 | No |
| 3 | SCG2 |  |  | 166 | 0.470 | 0.0408 | No |
| 4 | SLC38A1 |  |  | 992 | 0.212 | -0.0440 | No |
| 5 | SOX11 |  |  | 1197 | 0.185 | -0.0602 | No |
| 6 | KIF5C |  |  | 1422 | 0.160 | -0.0795 | No |
| 7 | ZFHX3 |  |  | 2308 | 0.091 | -0.1755 | No |
| 8 | HAND2 |  |  | 3418 | 0.031 | -0.2987 | No |
| 9 | MAPT |  |  | 4051 | 0.005 | -0.3694 | No |
| 10 | RTN1 |  |  | 4556 | -0.016 | -0.4253 | No |
| 11 | CD24 |  |  | 4590 | -0.018 | -0.4283 | No |
| 12 | KIF21A |  |  | 4843 | -0.027 | -0.4556 | No |
| 13 | NREP |  |  | 5261 | -0.043 | -0.5008 | No |
| 14 | COX6A1 |  |  | 5281 | -0.043 | -0.5014 | No |
| 15 | ENO2 |  |  | 5895 | -0.069 | -0.5676 | No |
| 16 | HSP90AB1 |  |  | 6611 | -0.109 | -0.6438 | No |
| 17 | HSP90AA1 |  |  | 6962 | -0.134 | -0.6782 | No |
| 18 | CRMP1 |  |  | 7368 | -0.177 | -0.7172 | No |
| 19 | CCT3 |  |  | 7464 | -0.187 | -0.7211 | No |
| 20 | MAP1B |  |  | 7666 | -0.213 | -0.7360 | No |
| 21 | HDAC2 |  |  | 7865 | -0.247 | -0.7492 | Yes |
| 22 | OCIAD2 |  |  | 7875 | -0.249 | -0.7413 | Yes |
| 23 | RBP1 |  |  | 7877 | -0.250 | -0.7323 | Yes |
| 24 | SOX4 |  |  | 7958 | -0.268 | -0.7317 | Yes |
| 25 | HMGB2 |  |  | 7973 | -0.271 | -0.7235 | Yes |
| 26 | TAGLN3 |  |  | 8037 | -0.283 | -0.7203 | Yes |
| 27 | TMSB15A |  |  | 8046 | -0.284 | -0.7110 | Yes |
| 28 | ATP6V0E2 |  |  | 8103 | -0.298 | -0.7065 | Yes |
| 29 | CDK6 |  |  | 8156 | -0.310 | -0.7011 | Yes |
| 30 | CBX1 |  |  | 8222 | -0.331 | -0.6964 | Yes |
| 31 | TUBB2B |  |  | 8224 | -0.332 | -0.6846 | Yes |
| 32 | CCND1 |  |  | 8346 | -0.368 | -0.6848 | Yes |
| 33 | TUBB2A |  |  | 8364 | -0.376 | -0.6732 | Yes |
| 34 | CKB |  |  | 8448 | -0.407 | -0.6678 | Yes |
| 35 | PAFAH1B3 |  |  | 8532 | -0.448 | -0.6609 | Yes |
| 36 | RAN |  |  | 8555 | -0.463 | -0.6467 | Yes |
| 37 | HNRNPA1 |  |  | 8570 | -0.469 | -0.6313 | Yes |
| 38 | ZNF428 |  |  | 8602 | -0.493 | -0.6170 | Yes |
| 39 | HMGN2 |  |  | 8608 | -0.496 | -0.5997 | Yes |
| 40 | JPT1 |  |  | 8647 | -0.523 | -0.5850 | Yes |
| 41 | MLLT11 |  |  | 8695 | -0.555 | -0.5703 | Yes |
| 42 | UCHL1 |  |  | 8700 | -0.565 | -0.5503 | Yes |
| 43 | FSCN1 |  |  | 8710 | -0.573 | -0.5307 | Yes |
| 44 | YWHAQ |  |  | 8730 | -0.592 | -0.5114 | Yes |
| 45 | RPL38 |  |  | 8741 | -0.598 | -0.4909 | Yes |
| 46 | CALM2 |  |  | 8744 | -0.600 | -0.4695 | Yes |
| 47 | TUBB |  |  | 8763 | -0.621 | -0.4491 | Yes |
| 48 | H2AFZ |  |  | 8774 | -0.630 | -0.4275 | Yes |
| 49 | STMN1 |  |  | 8783 | -0.638 | -0.4053 | Yes |
| 50 | RPS7 |  |  | 8791 | -0.644 | -0.3829 | Yes |
| 51 | MZT2B |  |  | 8816 | -0.665 | -0.3615 | Yes |
| 52 | FABP5 |  |  | 8829 | -0.684 | -0.3382 | Yes |
| 53 | BEX1 |  |  | 8833 | -0.689 | -0.3137 | Yes |
| 54 | RPL27 |  |  | 8840 | -0.698 | -0.2892 | Yes |
| 55 | H3F3B |  |  | 8846 | -0.705 | -0.2643 | Yes |
| 56 | HIST1H4C |  |  | 8850 | -0.710 | -0.2390 | Yes |
| 57 | SUMO2 |  |  | 8852 | -0.712 | -0.2134 | Yes |
| 58 | H3F3A |  |  | 8867 | -0.728 | -0.1887 | Yes |
| 59 | TUBA1A |  |  | 8883 | -0.751 | -0.1632 | Yes |
| 60 | GAP43 |  |  | 8890 | -0.759 | -0.1365 | Yes |
| 61 | PTMA |  |  | 8894 | -0.764 | -0.1093 | Yes |
| 62 | CHCHD2 |  |  | 8898 | -0.770 | -0.0818 | Yes |
| 63 | PPIA |  |  | 8913 | -0.797 | -0.0546 | Yes |
| 64 | GAPDH |  |  | 8915 | -0.803 | -0.0257 | Yes |
| 65 | HINT1 |  |  | 8961 | -0.923 | 0.0026 | Yes |
Table: GSEA details [plain text format]

  

Fig 2: OLSEN\_ADRENERGIC: Random ES distribution      
 Gene set null distribution of ES for **OLSEN\_ADRENERGIC**
